# PROFILE AND APPROPRIATE USE OF ANTIBIOTICS AMONG CHILDREN IN A GENERAL HOSPITAL IN SOUTHERN BRAZIL

**DOI:** 10.1590/1984-0462/;2019;37;1;00011

**Published:** 2018-07-26

**Authors:** Fernanda EmyInumaru, André Souza e Silva, Alessandra de Sá Soares, Fabiana Schuelter-Trevisol

**Affiliations:** a Universidade do Sul de Santa Catarina, Tubarão, SC, Brasil.; b Hospital Nossa Senhora da Conceição, Tubarão, SC, Brasil.

**Keywords:** Antibiotics, Child, Hospitalization, Medications errors, Antibacterianos, Criança, Hospitalização, Erros de Medicação

## Abstract

**Objective:**

To examine the profile and appropriate use of antibiotics among hospitalized children.

**Methods:**

A cross-sectional study was conducted with children who had taken antibiotics during hospitalization in a private philanthropic hospital in Southern Brazil, from January to December 2015. The data were obtained by reviewing medical records, encompassing demographic data (age, gender, ethnicity, and body weight) and clinical data (causes of hospitalization, use of antibiotics, and clinical outcome). Descriptive statistics was used to present the data.

**Results:**

Of the 318 participants included in the study, 61.3% were male patients. The age range varied between 2 and 11 years, with mean age of 5.8±2.9 years. The prevalence of antibiotics was 24.4% out of the 1,346 hospitalized children. Median hospital stay was four days. The main cause of hospitalization was clinical instability, and the most commonly prescribed antibiotics was Cefazolin, mostly administered intravenously. Regarding the administration of antibiotics, 62.2% were adequately prescribed, even though underdose was 11.7%, and overdose was 14.6% in the studied patients. Antibiotic administration intervals were characterized as long in 8% of cases, and short in 3.5% of cases.

**Conclusions:**

Although the prevalence of antibiotics among hospitalized children was not that high, a considerable part of the sample presented inadequacy regarding the dosage and range of use. These data raise concerns about bacterial resistance and adverse reactions.

## RESUMO

**Objetivo:**

Verificar o perfil e a adequabilidade do uso de antibacterianos em crianças hospitalizadas.

**Métodos:**

Estudo transversal. Foi feita a análise de todas as crianças que utilizaram antibacterianos durante a internação de janeiro a dezembro de 2015, em um hospital filantrópico de direito privado de grande porte no sul do Brasil. As informações foram obtidas por revisão dos prontuários e incluíram dados demográficos (idade, sexo, raça e peso corporal) e clínicos (motivo da internação, uso deantibacterianos e desfecho clínico). Utilizou-se estatística descritiva.

**Resultados:**

Dos 318 pacientes incluídos, 61,3% eram do sexo masculino. A faixa etária variou de 2 a 11 anos (média: 5,8±2,9 anos de idade). A prevalência do uso de antibacterianos foi de 24,4% considerando o total de 1.346 crianças que foram hospitalizadas. O tempo de internação apresentou mediana de quatro dias. O principal motivo de internação foi clínico e o antibacteriano mais prescrito foi a cefazolina, sendo a via intravenosa predominante. Em relação ao uso de antibacterianos, 62,2% apresentaram prescrições de antibacterianos consideradas adequadas. A subdosagem e a superdosagem tiveram, respectivamente, os valores de 11,7 e 14,6% dos pacientes incluídos. Quanto aos intervalos de administração, 8% foram caracterizados com intervalos longos e 3,5%, curtos.

**Conclusões:**

Apesar de a prevalência encontrada do uso de antibacterianos nas crianças hospitalizadas não ser tão elevada, parte considerável da amostra apresentou inadequabilidade quanto ao uso desse tipo de medicamento, se considerados a dose e o intervalo de utilização. Esses dados são motivo de preocupação para o desenvolvimento de resistência bacteriana e ocorrência de reações adversas.

## INTRODUCTION

The use of antibiotics by patients hospitalized in the pediatrics sector shows the importance of the treatment of infectious problems and conditions.^1^ Two to three infectious episodes may manifest in early childhood per year.^1^ A systematic review showed that the antibiotics represent the most prescribed therapeutic class in the pediatric age group.^2^ The children use twice as many antimicrobials as adults, so the amount of the prescription of this type of medication for the age group lower than 5 years is relevant.^3^ For pediatric patients, it is essential to know about the factors that can act on the response to medication, such as the toxicity of the drugs, the patient’s age, the presence of kidney and/or liver failure and possible drug interactions. These factors influence the pharmacokinetic and pharmacodynamic changes resulting from the physiological development at this age group.^4^

The child’s body metabolizes the medication differently from adults, once the children have relevant specificities, with differences regarding body composition and the presence of biochemical-physiological specificities that may interfere in the processes of absorption, distribution, metabolism and elimination of medications.^4^ Therefore, during the prescription of a drug, it is necessary to consider the physiological differences of the pediatric age group.^3^ ^,^ ^4^ Currently, there is no control policy over the record and prescription of medications to pediatrics, and as little medication prescription as possible is defended for hospitalized children.^5^ For the pediatric age group, there are only few clinical trials during the process of developing a new drug, once the following must be respected: ethical, legal and economic parameters. Therefore, the effects that these drugs can cause on the children will only be verified after its use in clinical practice, after the pharmaceutic record of the product.^5^

In 2017, the Ministry of Health published a document about Pharmaceutic Care in Pediatrics, which recommends several actions for the rational use of drugs in childhood. The document focuses on the need for clinical and epidemiological studies involving children, in order to guarantee the efficacy, safety and effectiveness of the drug treatment for this population. Besides, it points to the need for developing products for children, aiming at solving the neglected conditions, and for increasing the comfort and adherence to pharmacotherapy. It also suggests the inclusion of pediatric medications in the List of Essential Drugs (RENAME), and the need for a specific regulatory process in pediatrics.^5^

In this context, the objective of this study was to verify the profile and adequacy of the use of antibiotics among children hospitalized in different sectors of a hospital in Southern Brazil in 2015.

## METHOD

This is a cross-sectional epidemiological study which analyzed the use of antibiotics among children hospitalized in a large hospital in Southern Brazil from January to December 2015. The hospital is a philanthropic institution, and 80% of the services are carried out by the Unified Health System (SUS). The hospital is the largest on in terms of number of beds in the state of Santa Catarina, accounting for 410.

The study included all children aged between 2 and 11 years hospitalized in the aforementioned service who used antibiotics in the studied period. The sample was admitted to the sectors of Pediatrics and the Pediatric Intensive Care unit. Children aged less than 2 years were excluded, considering there were premature newborns and children with congenital malformations who had been hospitalized since birth. The variables of interest were sex, age, ethnicity, city of residence, medical diagnosis for hospitalization (International Code of Diseases - ICD-10) and discharge. The discharge ICD (discharge diagnosis ) was used because, during hospitalization, the diagnosis could be changed after the conduction of examinations. The variables related with the treatment were: prescribed antibiotics, route of administration, dosage and treatment interval.

For the presentation of data, the ICDs were grouped in clinical and surgical. Surgical were the patients who underwent, at any time, a surgical procedure, and sometimes the use of antibiotic prophylaxis. 

The adequacy of the dosage was assessed in patients in which body weight was available in the chart to calculate the correct dosage. Adequacy was classified in: adequate dosage, which considered the dosage according to the child’s weight; and inadequate dosage, subdivided in under dose or overdose, besides long (increasing time between dosages) and short intervals (reduced time of administration between doses). For that definition, the pharmacotherapeutic instructions of antibiotics contained in the parameters established in the International Drug Information Handbook was used for clinical purposes.^6^ The evaluation of adequacy of cases of surgical antibiotic prophylaxis required the protocols recommended by the Commission of Hospital Infection Control (CCIH) of the hospital.

Based on the screening regarding the prescription of antibiotics, made by the sector of Information Technology, the information was obtained by the review of patients’ charts, via the Tasy^®^ system, software used to electronically register the charts. The collected data were typed in the Microsoft Office Excel 2007 (Microsoft Corporation), and the statistical analyses were carried out in the Statistical Package for the Social Sciences (SPSS) v.21.0 (IBM, Armonk, New York, USA). The quantitative variables were described as measures of central tendency and dispersion, and the qualitative variables were described in absolute numbers and proportions.

This study was approved by the Research Ethics Committee of Universidade do Sul de Santa Catarina (CEP-Unisul), CAAE 55657416.9.0000.5369, report n. 1.527.036, on May3, 2016.

## RESULTS

During the study period, 329 participants were selected based on the hospital’s report. However, 11 charts were excluded for not presenting the variables of interest of the study, resulting in a 3% loss. Therefore, the final sample was comprised of 318 participants. The prevalence of the use of antibiotics was 24.4%, considering the total of 1,346 hospitalized children in 2015. The age group of the participants ranged between 2 and 11 years, with mean age of 5.8±2.9 years. The time of hospitalization presented a 4-day median, ranging from 0 to 60 days of hospitalization. Table 1 presents the sociodemographic and clinical data of the study participants.

**Table 1:** Profile of sociodemographic and clinical data of hospitalized children using antibiotics in 2015 (n=318).

	n	%
Age (years)		
2-5	162	50.9
6-9	114	35.9
10-11	42	13.2
Sex		
Female	123	38.7
Male	195	61.3
Ethnicity		
Caucasian	287	90.3
Non-caucasian	23	7.2
Not informed	8	2.5
City of residence		
Tubarão	168	52.9
Other cities of Santa Catarina	149	46.8
Other states	1	0.3
Reason for hospitalization		
Clinical	184	57.9
Surgical	134	42.1
Time of hospitalization (days)		
0-1	60	18.9
2-4	126	39.6
5-7	61	19.2
8-15	49	15.4
>15	22	6.9
Outcome		
Death	2	0.6
Discharge	309	97.2
Transfer	7	2.2

Source: Hospital Nossa Senhora da Conceição, Tubarão, Santa Catarina, 2015.

As to the reason for hospitalization, defined by the ICD, among the clinical diagnoses, the respiratory tract diseases stood out and came first; bacterial pneumonia and acute tonsillitis were the most common ones. Then came the genitourinary tract conditions, such as chronic non-obstructive pyelonephritis, associated with reflux and other urinary tract disorders; in third place came the skin and subcutaneous tissue conditions, represented by cellulitis and skin abscess. Regarding surgical diagnoses, first came the gastroenterological surgeries, represented by appendicectomies, mostly; then came the orthopedic surgeries, mostly femur fracture repair; and in third came the genitourinary surgeries, especially hypospadias and postectomy.

**Figure 1:**
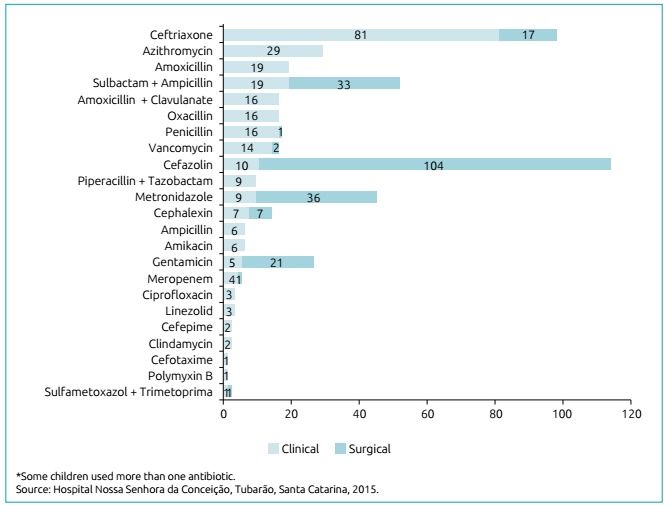
Frequency of antibiotics used in hospitalized children in 2015 (n=502).

Figure 1 represents the antibiotics prescribed for systemic use. Besides the 502 of systemic use, 156 were topic antibiotics, accounting for 658 prescriptions of this type of drug. Considering this number, regarding the route of administration, 416 (63.3%) were had venous administration, 156 (23.6%), topical administration; 76 (11.6%), oral administration; and 10 (1.5%), intramuscular administration. The Topical antibiotics used were: chloramphenicol, neomycin sulfate and bacitracin, and 1% silver sulfadiazine

Table 2 presents the adequacy of the use of antibiotics. Regarding such adequacy, in the 179 children in which it was possible to calculate the ratio between the dosage and the interval prescribed according to weight, 4 patients presented not only with underdose, but also short intervals, and 14 patients being underdosed had associated long intervals. Besides, there were two cases of overdose allied with short intervals, and four overdose cases associated with long intervals. There was more inadequacy in the use of antibiotics among surgical patients with compared to clinical cases (*p*=0,014).

**Table 2:** Adequacy of the use of antibiotics prescribed for hospitalized children in 2015.

Adequacy of the antibiotic*	Clinical use (n=253)	Surgical use (n=62)	Total
Adequate	160 (63.2%)	36 (58.1%)	196
Underdose	26 (10.3%)	11 (17.7%)	37
Overdose	42 (16.6%)	4 (6.5%)	46
Short intervals	9 (3.6%)	2 (3.2%)	11
Long intervals	16 (6.3%)	9 (14.5%)	25

*Adequacy was carried out in cases in which there was a description of the posology of the antibiotic and the children’s weight to calculate the dosage. Values are expressed in n (%). Source: Hospital Nossa Senhora da Conceição, Tubarão, Santa Catarina, 2015.

## DISCUSSION

This study showed that more than half of the antibiotic prescription was adequate according to the literature used as reference.^6^ The adequacy of the use of antibiotics is essential to ensure the resoluteness of the infection, preventing bacterial resistance and adverse reactions to drugs.^7^ When beginning the pharmacological treatment of an infection, the choice of the type of antibiotic to be prescribed (related with its efficacy and safety), the route of administration and the duration of the treatment (related with the comfort in the use of the drug) can be responsible for therapeutic success, based on the precepts of the rational use of medications.^7^

The prevalence in the use of antibiotics in the study period was 24.4%, similarly to the result found by Tonello et al*.* - 25.7%.^8^ As in this study, these authors assessed the use of medications among children aged up to 11 years in a pediatric unit of a private hospital in the South of Brazil. However, Zavala-González and Sánchez-Santana evaluated the quality of the prescription of antibiotics in a pediatric service in a hospital in Mexico, and found prevalence of use of 80.3% among 152 children aged from 0 to 14 years, which is much higher to the data observed in this study.^9^

In the analyzed sample, 38.4% of the patients using antibiotics presented inadequacy as to the prescribed posology, and this percentage was lower to that found by other studies, being more common among surgical patients. In the study by Zavala-González and Sánchez-Santana, 93% of the antibiotic prescriptions were inadequate.^10^ Inadequacy can result from the lack of specific policies for the pediatric age group regarding the prescription of antibiotics.^11^ It is important to mention that in the hospital where this study was carried out, the use of antibiotics follows the protocols and recommendations developed by CCIH for the use of antibiotics in pediatrics. In spite of that, the protocols of the service shows no de-escalation schedule, which is a relevant item for the prevention of unwanted reactions in the pediatric patient, besides cost reduction. Antibiotic de-escalation is defined as the narrowing of the antimicrobial spectrum guided by the susceptibility of the pathogen, reducing the chances of creating bacterial resistance. As soon as possible, it is important to narrow the antibiotic spectrum, considering the clinical condition of the patient, the pathogens identified in the results of the cultures, and the profile of sensitivity shown by the antibiogram; besides, when there is no evidence of bacterial infection, it is recommended to suspend the antibiotic.^12^ The difficulty of this schedule in the study location is owed to the occurrence of the clinical diagnosis in most pediatric infections.

Among the inadequacies found in this study, the highest prevalence was overdose (14.6%). Pereira and Bezerra also found this result, but with higher frequency (53.9%).^13^ The overdose of antimicrobials in children may lead to toxicity and increasing mortality rates.^14^ Besides, the lack of adequate medications in pediatrics leads to the need for fractioning the dose, in order to adapt it to the pediatric age group, which can compromise the safe use of the drug. A study by Gonçalves et al., which analyzed the use of systemic antimicrobials in children and adolescents in two hospitals, observed that the lower the pediatric age group, the higher the inadequacy of the drug, requiring dosage adaptation.^15^ The hospitalized pediatric patients presented with higher risk of adverse effects related with errors in medication, once this population is subjected to dosage errors and/or the incorrect selection of the therapeutic class to be prescribed.^16^ According to Girotto and Silva, the inadequate use of antibiotics can increase the costs with hospitalization due to the longer period of hospitalization, to the non-resolution of the clinical situation or to the intercurrence of adverse events, such as intoxication or hypersensitivity reactions.^17^

This study verified that, in 11.7% of the cases, the antibiotics were underdosed. Tonello et al*.* observed underdose in the following antibiotics: ampicillin, gentamicin and penicillin.^8^ The use of piperacillin + tazobactam among infants presented higher frequency of underdose in the study by Girotto and Silva.^18^ The administration of doses lower to recommended may lead to therapeutic flaw, and contribute with the onset of bacterial resistance, once there is a reduction in the efficient plasmatic concentration of the antibiotic.^11^ Bacterial resistance is, therefore, related with inadequate therapeutic treatment, be it because of underdose, prolonged use or selection of an inefficient antibiotic selection for the etiological agent of the infection.^19^ Other factors have benefitted the increasing bacterial resistance; among them, the inadequate and large-scale use of antibiotics stands out, especially in community infections, added to the lack of prophylactic measures, immunological deficiency and the delayed definitive diagnosis.^20^

Another factor analyzed in this study was that 11.5% of the prescriptions contained errors regarding the interval of the dose, which represents a risk due to the medication’s half-life. The administration of inadequate antibiotic intervals can also induce to therapeutic flaw and bacterial resistance.^21^ Regarding the study of Paganotti et al., it was observed that all of the prescribed antibiotics in relation to the posology intervals were in accordance with literature.^22^ This can be owed to the more strict control and supervision of the distribution of antibiotics based on the established protocols.

During the study, cefazolin represented the most frequently used antibiotic. One of the reasons for this result is the use of this drug for purposes of surgery prophylaxis, according to the recommendations of CCIH in the studied hospital, considering that 42.1% of the patients were hospitalized for surgical reasons. This finding is in agreement with the study by Ferreira et al., which found cefazolin was the most prescribed antibiotic in a hospital environment.^23^ The second most frequent antiobiotic prescribed is ceftriaxone, similarly to the findings by Ralph et al*.* ^24^ This result is explained by the broad spectrum aspect of this medication, for being a third generation cephalosporin, which can reach gram-negative bacteria and transpose the hematoencephalic barrier, with indication of use among hospitalized patients.^24^ In this study, only one patient used polymyxin B, antibiotic used for gram-negative bacterial infections which acquired resistance to other drugs. However, due to its possible nephrotoxic and neurotoxic effects, its use must be monitored, especially in children.^25^

This study also observed that the venous route was more frequent for the administration of the medicine. Other studies also verified the preference for the parenteral route.^11^ ^,^ ^26^ ^,^ ^27^ Considering that one of the factors to determine the route of administration of the antibiotic is the severity of the infection, the same result may result from the fact that the studies involved children treated in a hospital environment for more severe infections, or for surgical antibiotic prophylaxis. Therefore, the indication for the intravenous route.^27^

Regarding the study limitations, the prescriptions of antibiotics were based on the patient’s clinic, once laboratory or bacterial culture criteria with isolation of the pathogen and respective sensitivity test to antimicrobials to assess the adequacy of the prescription were not always found. We did not analyze biological markers, such as procalcitonin, to assess the resoluteness of the infection. It was not possible to calculate the adequacy of the dose for all patients in the studied sample, once there were no records of body weight in all charts of the children included in the study. This may have occurred due to the flaw in the transmission of patients’ data from the physical to the electronic chart, or even the non-assessment of that item. The lack of this data may compromise patients’ safety, once the chances of dose inadequacy are higher. Since this is a cross-sectional study, the data were assessed at a specific moment, without following-up the time of treatment.

Based on the data of this study, it was possible to conclude that even though the prevalence of the use of antibiotics in hospitalized children is not that high, a considerable part of the sample presented with inadequacy regarding the use of this class of medications, considering the dose and the interval of use of this class of drugs. These data are a reason of concern for the development of bacterial resistance and for the occurrence of adverse reactions in this population.
