# ERRATUM

**DOI:** 10.1590/1984-0462/;2019;37;1;00011erratum

**Published:** 2018

In the manuscript “Profile and appropriate use of antibiotics among children in a general hospital in Southern Brazil”, DOI: 10.1590/1984-0462/;2019;37;1;00011, published in the Rev. paul. pediatr. July 26, 2018. [Epub ahead of print]

Where it reads:

^a^Universidade do Sul de Santa Catarina, Palhoça, SC, Brazil.

^b^Hospital Nossa Senhora da Conceição, Porto Alegre, RS, Brazil.

It should read:

^a^Universidade do Sul de Santa Catarina, Tubarão, SC, Brazil.

^b^Hospital Nossa Senhora da Conceição, Tubarão, SC, Brazil.

